# Personalized Nutrition—Genes, Diet, and Related Interactive Parameters as Predictors of Cancer in Multiethnic Colorectal Cancer Families

**DOI:** 10.3390/nu10060795

**Published:** 2018-06-20

**Authors:** S. Pamela K. Shiao, James Grayson, Amanda Lie, Chong Ho Yu

**Affiliations:** 1 College of Nursing and Medical College of Georgia, Augusta University, Augusta, GA 30912, USA; 2 Hull College of Business, Augusta University, Augusta, GA 30912, USA; jgrayson@augusta.edu; 3 Citrus Valley Health Partners, Foothill Presbyterian Hospital, Glendora, CA 91741, USA; amandalie413@gmail.com; 4 School of Business, University of Phoenix, Pasadena, CA 91101, USA; alexyu@email.phoenix.edu

**Keywords:** gene-diet interaction, colorectal cancer, predictor, multiethnic groups

## Abstract

To personalize nutrition, the purpose of this study was to examine five key genes in the folate metabolism pathway, and dietary parameters and related interactive parameters as predictors of colorectal cancer (CRC) by measuring the healthy eating index (HEI) in multiethnic families. The five genes included *methylenetetrahydrofolate reductase* (*MTHFR*) 677 and 1298, *methionine synthase* (*MTR*) 2756, *methionine synthase reductase* (*MTRR* 66), and *dihydrofolate reductase* (*DHFR*) *19bp*, and they were used to compute a total gene mutation score. We included 53 families, 53 CRC patients and 53 paired family friend members of diverse population groups in Southern California. We measured multidimensional data using the ensemble bootstrap forest method to identify variables of importance within domains of genetic, demographic, and dietary parameters to achieve dimension reduction. We then constructed predictive generalized regression (GR) modeling with a supervised machine learning validation procedure with the target variable (cancer status) being specified to validate the results to allow enhanced prediction and reproducibility. The results showed that the CRC group had increased total gene mutation scores compared to the family members (*p* < 0.05). Using the Akaike’s information criterion and Leave-One-Out cross validation GR methods, the HEI was interactive with thiamine (vitamin B1), which is a new finding for the literature. The natural food sources for thiamine include whole grains, legumes, and some meats and fish which HEI scoring included as part of healthy portions (versus limiting portions on salt, saturated fat and empty calories). Additional predictors included age, as well as gender and the interaction of *MTHFR* 677 with overweight status (measured by body mass index) in predicting CRC, with the cancer group having more men and overweight cases. The HEI score was significant when split at the median score of 77 into greater or less scores, confirmed through the machine-learning recursive tree method and predictive modeling, although an HEI score of greater than 80 is the US national standard set value for a good diet. The HEI and healthy eating are modifiable factors for healthy living in relation to dietary parameters and cancer prevention, and they can be used for personalized nutrition in the precision-based healthcare era.

## 1. Introduction

Chronic inflammation is a major risk factor for colon and rectum health for the prevention of colorectal cancer (CRC) [1,2,3,4,5,6]. CRC is the number one most preventable cancer for men and women in the world [7]. The most significant contributing factors in CRC development have been recognized as preventable as they are associated with environmental and lifestyle factors, rather than being inheritable factors [8,9,10]. Therefore, cultivating healthy lifestyles and healthy eating can help prevent CRC through epigenetic mechanisms [11]. Recent studies have documented gene-environment interactions and the development of various diseases including CRC [12,13,14,15,16] through oxidative stress pathways [17,18,19]. Deficiencies in macro and micronutrients, such as folate and B-vitamins, as methyl-donors can contribute to the impairment of the one-carbon metabolism (OCM) pathway which may lead to CRC [20,21,22,23]. Hence, genes, diet, and interactive parameters involved in inflammatory processes related to CRC are worthy of investigation, particularly when a poor diet is combined with excess caloric intake, weight gain, and unhealthy practices, such as smoking and overconsumption of alcohol, which increase inflammatory responses [24,25,26,27].

The *methylenetetrahydrofolate reductase* (*MTHFR*) gene affects MTHFR, a key enzyme in folate metabolism [28,29]. It irreversibly catalyzes the conversion of 5,10-methylene tetrahydrofolate (MTHF) to 5-MTHF or methyl folate, the primary circulatory form of folate and a carbon donor for the remethylation of homocysteine to methionine. *Methionine synthase* (*MTR* A2756G, rs1805087) secretes the MTR enzyme, requiring methylcobalamin (methyl B12) for its activity, and catalyzes the remethylation of homocysteine to methionine. Furthermore, *methionine synthase reductase* (*MTRR* A66G, rs1801394) polymorphisms increase homocysteine levels [30,31,32]. *MTRR* produces an enzyme that activates cobalamin-dependent methionine synthase for the biosynthesis of methionine, as a precursor for methylation reactions, and to regenerate nucleotide biosynthesis [33,34,35,36]. In addition, *dihydrofolate reductase* (*DHFR*) (19 base pairs (19 bp), rs70991108) catalyzes the reduction of dihydrofolate to tetrahydrofolate (THF) and plays an essential role in cellular metabolism and growth by shuttling the methyl group with the use of THF to allow the synthesis of essential metabolites [32,37]. Mutations on *MTHFR* 677 (rs1801133, homozygote 677TT with 70% and heterozygote 677CT with 35% loss of function) and *MTHFR* 1298 (rs1801131, homozygote 1298CC with 30% and heterozygote 1298AC with 15% loss of enzymatic function) increase plasma homocysteine levels [30,31]. Therefore, gene polymorphisms in the OCM pathway can decrease supplies of metabolites and cofactors, such as folate and B-vitamins, increasing the risk of CRC. Nutrients that can act as methyl donors related to these genes, including folate (vitamin B9) and vitamin B12, play integral roles in the phenotypic expression of related gene mutations in methylation pathways [28,29,30,31,38,39].

A healthy diet is generally classified as a high intake of fruits and vegetables, wholegrains, nuts and legumes, fish and other seafood, and milk and other dairy products, and decreases the risk of CRC [1,2,24,25,26,27]. Additionally, healthy eating involves limiting salt, saturated fat, and empty calories from sugar and alcohol as additional dietary parameters [24,25,26,27]. The American Institute for Cancer Research (AICR) found strong evidence that the following factors decrease CRC risk: Eating more plant-based foods in addition to maintaining a healthy weight, and reducing red meat and alcohol intake [7,40]. One study found that participants who followed 4–6 of these recommendations over the course of 8 years decreased their risk of developing CRC by half [41]. Various methods have been created to score overall dietary patterns, a well-known method being the Health Eating Index (HEI) [42,43,44,45,46]. Case-control [47,48] and cohort [49] studies have shown that greater HEI scores are associated with lower CRC risk. In regard to potential gene–diet interactions [50,51,52], diets rich in fiber, folate as a methyl donor, and calcium, with limited pro-inflammatory fatty acids are associated with protective effects against CRC. A compilation of gene mutations in the OCM pathway were noted to be associated with potential gene-diet/environment interactions related to CRC risk and prevention [36,53,54,55].

In summary, lifestyles including healthy dietary habits that potentially interact with genetic factors are important considerations for personalized nutrition in the precision-based healthcare era [36,53,54,55]. Family members can be involved in CRC prevention [8,9,10] by providing additional evidence for prevention efforts for cancer prevention. Therefore, following a previous report on gene-environment interactions related to CRC prevention [55], we examined genes with dietary, demographic, and interactive parameters in association with the risk of CRC in diverse family-based ethnic groups. We measured multidimensional data using the ensemble method [56,57,58,59] to identify variables of importance within domains of genetic, demographic, and dietary parameters. We then constructed predictive generalized regression (GR) modeling with a supervised machine learning validation procedure with the target variable (cancer status) being specified to validate the results for enhanced prediction and reproducibility [60,61,62,63]. 

## 2. Materials and Method

### 2.1. Study Population and Setting

A total of 106 human subjects participated and completed dietary data instruments, 53 CRC and 53 paired family/friend members. We accessed the California Cancer Registry (CCR) database and additional cases through case referrals by the participants. The human subjects protocol was approved by the designated appropriate Human Subjects Institutional Review Boards (IRB) from the California State Committee for the Protection of Human Subjects for data access through the CCR (CPHS-12-12-1007, approved 2013–2019) and from the local educational institutions (Azusa Pacific University, approved 2013–2015; Augusta University, 806069-7, approved 2015–2018) [55] (see Supplementary file for Informed Consent Form). Inclusion criteria has been reported previously [55] and is summarized as follows: participants had to (a) be expected to live for at least 6 months; (b) be 18–80 years of age; (c) have a family/friend member nearby to act as the case and family/friend pair, (d) have adequate cognitive and mental capacities, (e) be willing to participate in the interviews and biological samples for the genotyping data collection. 

### 2.2. Demographic and Genetic Measurements 

The measurements and instruments used in this study have been reported previously [55], including the health-related lifestyle and dietary status [64], family history, functional capacities, cancer risks and activities, demographics [65], and family pedigrees (www.nchpeg.org) [66]. The five genes in the folate metabolism-related pathway included in the study were the *MTHFR* gene polymorphisms, C677T (rs1801133) and A1298C (rs1801131) involved with MTHFR enzymes which elevate homocysteine levels [28,29,30,31]; *DHFR* 19 base pairs (19 bp) (rs70991108) which are involved in folic acid conversion into methylenetetrahydrofolate (MTHF), the usable folate form [32,33]; and *MTR* A2756G (rs1805087) and *MTRR* A66G (rs1801394) which convert/recycle homocysteine back to usable MTR for the methylation cycle [34,35,36,37]. Gene mutations of folate metabolism-related pathways could lead to the loss of functions related to the methylation process [55]. The total possible gene polymorphism rates of the five chosen genes in the folate methylation pathways ranged from 0 to a possible maximum score of 10 if each of the five genes had homozygous polymorphisms. The presence of an MTHFR enzyme deficiency was calculated by combining the loss of enzyme functions from *MTHFR* C677T (loss of 35% for each of the two T polymorphic alleles) and *MTHFR* A1298C (a loss of 15% for each of the two C polymorphic alleles) to give a composite score of both *MTHFR* C677T and *MTHFR* A1298C polymorphisms [55,67]. Genotyping procedures have been described elsewhere earlier using the Taqman Technique [55,68,69].

### 2.3. Dietary Indexes

We used two tools to assess the dietary and nutrient intakes: The Healthy Eating Index (HEI-2015) [42,44] and recommended daily intakes (RDI) [43] which were collected with the Food Frequency Questionnaire (FFQ) [70,71] and data processed through the Nutrition Data Systems for Research (NDSR) [72,73]. The agreement and bias for the FFQ against NDSR has been reported before for this sample [74]. The correlations between the two measurements for the major caloric parameters ranged from 0.91 (fat, SE (standard error): 1.3%, −12 ± 15) to 0.95 (protein, SE: 0.76%, −17 ± 8.8); 0.86 for B9 (SE: 2.1%, −7.9 ± 0.2), and 0.99 for B12 (SE: 1.2%, −17 ± 0.1).

The HEI includes items of healthy portions of various quality food groups and limited portions of unhealthy food groups, issued by the US Department of Agriculture (USDA) based on the Dietary Guidelines for Americans (DGA) standards for a healthy lifestyle. The HEI is composed of 12 scored components which include the 5 major food groups—Fruit (total and whole), vegetables (total and greens/beans), grains (total and whole), dairy and protein and oils and nuts—In addition to limiting the intake of saturated fats, sodium, and empty calories. The total HEI score is the sum of the components and has a minimum score of 0 and a maximum score of 100. A score between 0–50 indicates a poor diet; 51–80 indicates a moderate diet quality that needs improvement; and a score greater than 80 indicates a good diet [42]. The recommended daily intakes (RDI) are issued by the Food and Nutrition Board of the Institute of Medicine which recommends the average daily levels of intake that are sufficient to meet the nutrient requirements of most healthy people based on gender and age [43]. Macronutrients include carbohydrates, protein, total and saturated fat, and cholesterol. Micronutrients include B-vitamins—(B9 (folate), B1 (thiamine), B2 (riboflavin), B3 (niacin), B6 and B12), vitamins A, C, D, and E, calcium, magnesium, iron, zinc, and methionine [75].

### 2.4. Data Analysis

The details of data analysis have been presented previously [55] and are summarized in the following text. We employed various methods, including the visualization and identification of data patterns related to family dependence [76], the ensemble method to identify variables of importance for the dimension reduction of multidimensional data, and predictive model building using JMP Pro 13 (SAS Institute, Cary, NC, USA) [77,78]. Influential predictors were identified using bootstrap forest prediction modelling in three categories: genetic, demographic and lifestyle, and dietary intake factors. Column contribution and variable importance were examined within each category. From the rank order of column contributions, the most influential variables were selected using the bootstrap forest method as variables of significance [56,57,58,59,77,78]. The column contribution was presented using *G*^2^ statistics for classification accuracy, which was derived from the conventional likelihood ratio *X*^2^ statistic. However, unlike *X*^2^ analysis, *G*^2^ results are not subject to sample size effects. *X*^2^ is a test of goodness-of-fit between the expected count and the actual count. By the same token, *G*^2^ indicates how well the expected count and actual count are classified into those groups. Ensemble methods included bootstrap forest and recursive trees [45,46,47,48], which are suited for small-sample studies [79], with a machine learning approach [80]. This has been shown to outperform single models, including regression or univariate statistics [81,82]. The misclassification rates of each model were compared to verify the function of a predictive model for the genetic, demographic, and dietary categories.

We then utilized GR with supervised machine learning validation, because the target variable had been specified, to obtain a smaller prediction error [77]. The index of complexity, Akaike Information Criterion with correction (AICc), was used [83,84,85,86,87] to test the fitness of the models, with smaller AICc values indicating optimal models. AICc outperformed the *R*^2^ and adjusted *R*^2^ methods which tend to favor complexity for the model quality [65]. We used the Elastic Net [88] and validation methods including the AICc validation and Leave-One-Out (LOO) cross validation methods due to their effectiveness on small data sets [89]. We assessed the model performance using the misclassification rate (smaller is better), AICc, and the area under the receiver operating characteristic (ROC) curve (AUC). The primary criterion was the fitness indicator with AICc to counteract the common problem in traditional statistics: overfitting. A well-predicted model might be an overfitted model, and thus, predictive accuracy is the secondary criterion and was determined using the misclassification rate and AUC.

GR is also known as penalized regression, meaning that the variable selection process penalizes complexity. To get the optimal model, the algorithm imposes a penalty on the model when redundant predictors are included. When there are several collinear predictors, least absolute shrinkage and selection operator (LASSO) selects just one and ignores others or zeroes out some regression coefficients. The Ridge method counteracts collinearity and variance inflation by shrinking the regression coefficients towards zero, but not exactly zero. The Elastic Net method combines the penalties of both the LASSO and Ridge approaches. While Lasso might shrink the coefficient of an unimportant variable all the way down to zero and Ridge just shrinks it towards zero, Elastic Net is in the middle, and thus, it tends to yield the most optimal model by balancing variance and bias. With the use of early stopping, Elastic Net is suitable for handling a data set with many variables and a few observations. In Elastic Net, a stage-wise algorithm called LARS-EN (least angle regression of Efron et al., 2004 [90,91] efficiently finds the best solution path. In short, it is more likely to balance variance and bias than other methods. Unlike linear least squares, when estimating the unknown parameters in a linear regression model, GR can simply zero out certain unused predictors [92,93,94,95]. In this case, the *p*-values in the linear regression model at most could only be 0.9999, but not exactly 1. However, when all permutations are exhausted, such as what is done in an exact test, the probability could be exactly 1. Along a similar vein, GR exhausts different paths to find the best model. When the full model has a mixture of important and unused predictors, the *p*-value cannot be 1. However, when the data can be perfectly described by the restricted model that results from path searching, the probability of observing the data can be 1. 

When developing a GR model for a predictive model, the first type of model presented in JMP Pro 13 is a logistic regression (LR) model, because it is the default estimation method. After this default method, other model launches can be pursued by choosing a variety of estimation methods (LASSO, Elastic Net, and others) and associated validation methods (a validation column, minimum AICc, LOO validation, and others) [90,96,97]. We chose the AICc validation and LOO cross validation methods because of their effectiveness for small data sets [98]. In effect, the default LR method could be characterized as an explanatory model, whereas the other GR estimation methods might best be characterized as predictive models. An explanatory model is typically used to explain the associations between the model parameters and the model response to test causal hypotheses, whereby a predictive model is used to predict future observations [99]. The nature of the model objectives (causal versus predictive) directly influence the underlying algorithms which can result in different results from models using the same set of initial parameters. Typically, using an explanatory model, the set of statistically significant parameters is identified for a final model. The predictive model using GR pursues methods to shrink coefficients towards 0, in part to guard against overfitting the model. For model prediction in GR analysis, continuous variables are recoded into new dichotomous variables, grouped by either median distribution or known score criteria, such as those related to healthy eating. 

The interactive prediction profilers were used to visualize the direction of association between two parameters (a predictor or factor with the outcome variable of healthy eating status or health outcomes in the profiler) or among three parameters (set of interactive variables with non-parallel distribution in addition to the outcome status of healthy eating or health outcomes in the interactive profilers). The visualization of the interactive profilers enables the analyst to ask “what-if” questions. Specifically, the analyst manipulates the levels of included variables to see how the model changes. By doing so, we can understand how the interaction of various factors affects the outcome and the sensitivity of the model.

## 3. Results 

### 3.1. Characteristics of Study Participants

Table 1 presents the comparisons of the key demographic factors between the control and cancer groups. The significantly different parameters between the control and cancer groups included gender, age, and total number of gene polymorphism mutations (all *p* < 0.05). We previously reported the distribution of the polymorphisms for the control and cancer groups and the four racial-ethnic subgroups [55] using the Hardy-Weinberg equilibrium (HWE) analysis. The total gene mutation score presented a median split between <4 and ≥4 for this sample and was significantly increased for the CRC group compared to the family/friend controls (*p* < 0.05) (Table 1).

The comparisons of demographic factors across the racial–ethnic subgroups are presented in Supplementary Table S1. Based on the body mass index (BMI) measurement, more than 50% of Hispanic and Black participants in this study were obese, a much greater proportion than in the White (29%) and Asian (0%) samples (*p* < 0.0001). More Whites in this study drank alcohol than the other three racial groups (*p* = 0.0007). In regard to the total gene mutation score on the five genes in the folate metabolism-related pathway, more Asian and White participants had greater total gene mutation scores than Hispanic and Black participants (see Supplementary Table S1).

### 3.2. Dietary Parameters

In regard to the comparisons of dietary parameters, no items were significantly different between the control and case groups in the HEI (Table 2) or RDI (Table 3). However, in terms of the differences in HEI parameters between racial groups, Asians had greater total fruit intakes (2.3 cups) and whole fruit intakes (1.6 cups) compared to the other three racial groups (both *p* < 0.001). Caucasians had the next highest fruit intakes (1.3 cups of total fruit and 1.01 cups of whole fruit), and Hispanics and African Americans had similarly low fruit intakes (see Supplementary Table S2). Another significant difference between racial groups was sodium intake (*p* < 0.05). While all racial groups consumed greater than the RDI levels for sodium, Asians had the highest sodium intake of 3.79 g, followed by Hispanics with 3 g, then Caucasians with 2.8 g, and African Americans with 2 g (see Supplementary Table S2). In the four racial groups, more than half of the sample ate more than 45% of the RDI for carbohydrates (*p* < 0.05), Asians having the highest intake (85%), followed by African Americans (77.8%), Hispanics (65.2%), and lastly, Caucasians (55.9%). Another significant dietary parameter was total fat. Hispanics had the highest intake (52.2%), consuming greater than 35% of their total calories from fat and exceeding the RDI, followed by Caucasians (47.1%), then African American (33.3%), and Asians (15%). In regard to the saturated fat intakes, more African Americans (77.8%), Caucasians (61.8%), and Hispanics (56.5%) consumed over the RDI for saturated fat than Asians (35%) (*p* < 0.05) (see Supplementary Table S3).

### 3.3. Most Influential Predictors of Variables of Importance 

Through the identification of the variables of importance, the most crucial predictors from the genetic, demographic, and dietary categories were identified. In terms of dietary parameters, all individual parameters involved in the HEI and RDI were tested. A HEI score of 77, the median split for this study sample, (instead of HEI 80) was used as the significant dietary predictor. The most crucial dietary variables of importance appeared in rank order (see Supplementary Table S4) as the total vegetable intake [10 ounce (oz)], followed by the total folate intake (100% RDI), vitamin B12 (150% RDI), total grains (4 oz), and HEI (median score 77). The most crucial genetic predictor was identified as the total number of gene polymorphism mutations (≥4) for all five genes combined. The significant demographic factors included gender and body weight. For all domains, Table 4 presents the rank order of the 10 predictors, including the demographic characteristics of age, gender, and overweight status (BMI status); two genetic parameters, including the total polymorphism score and MTHFR 677; and five dietary parameters, including the total vegetable intake (10 oz), total folate intake (100% RDI), HEI (score of 77), vitamin B12 (150% RDI), and thiamine (100% RDI). 

### 3.4. Predictors of Cancer from Genes, Diet, and Interactive Parameters 

**Figure 1:**
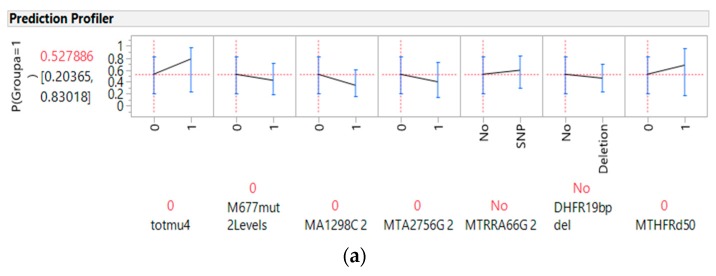

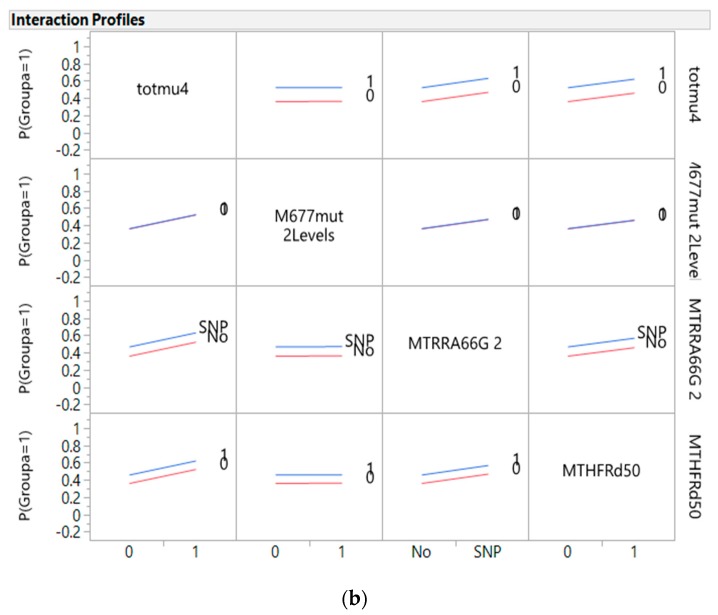
Genes involved in the prediction of colorectal cancer: (**a**) per single gene profiler, total polymorphism score and total methylenetetrahydrofolate reductase (MTHFR) enzyme deficiency (calculated based on *MTHFR* 677 and 1298 (presented as M677mut 2Levels and MA1298C 2) polymorphism mutation alleles), (**b**) interaction profilers of selected gene parameters and colorectal cancer. Note that the *MTHFR* 677 polymorphism status (0 or 1) overlapped with no discrimination against other genetic factors in regard to its association with cancer risk; *p*(Groupa) = 1 is the probability of predicting a level 1 (cancer status versus 0, the control status) response, MTA2756G 2: methionine synthase A2756G in 2 levels, MTRRA66G 2: methionine synthase reductase A66G in 2 levels, DHFR19bp del: dihydrofolate reductase 19 base pair deletion, MTHFRd50: MTHFR enzyme deficient 50% or higher.

Figure 1a further illustrates the profiler of the five genes, the MTHFR enzyme deficiency score and the total gene polymorphism mutation score in association with the CRC risk, and Figure 1b, shows examples of key interaction profiles of these gene parameters with the CRC risk. It is noteworthy to point out that while the *MTHFR* 677 and 1298 gene polymorphisms had downward trend associations with the CRC risk, the MTHFR enzyme deficiency score showed an upward or positive correlation with the CRC risk (Figure 1a). The interaction profilers for the associations of these seven gene parameters with CRC risk, as presented in Figure 1b, were all parallel lines, indicating no two-way interactions for these seven gene parameters in association with the CRC risk. 

**Figure 2:**
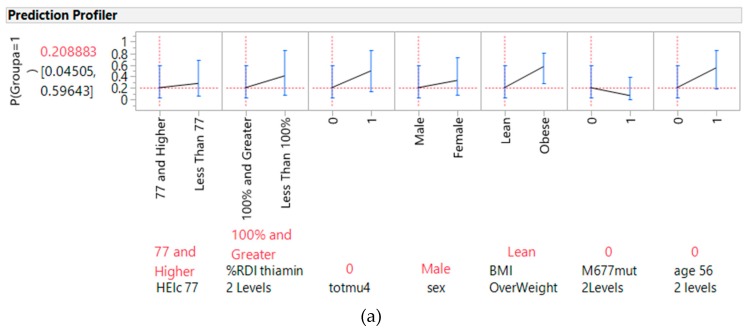

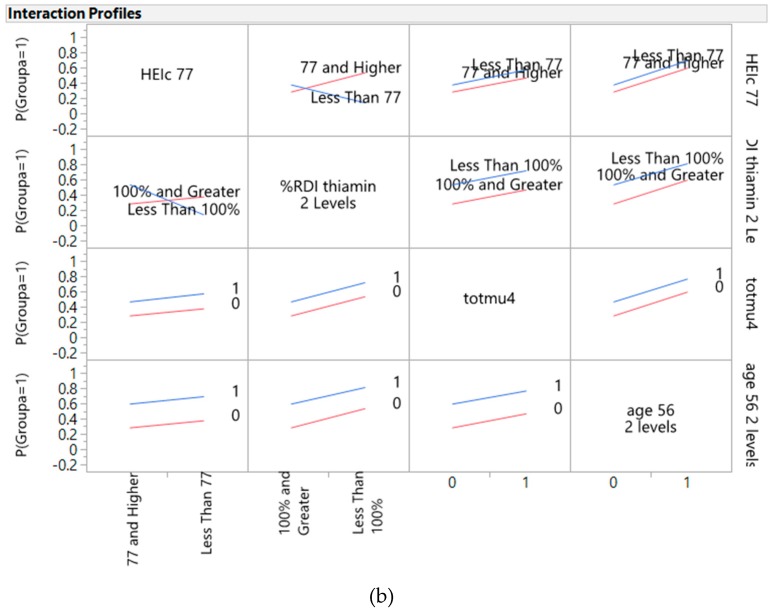
Gene-diet interactions relevant for the prediction of cancer: (**a**) prediction profiler, (**b**) interaction profiles (Healthy Eating Index interactions with with thiamine, with non-parallel lines for associations with cancer). Note: *p*(Groupa) = 1 is the probability of predicting a level 1 (cancer status versus 0, the control status) response.

Figure 2a present the profiler of HEI, thiamine, the total gene mutation score of the five genes, *MTHFR* 677 polymorphism mutations, overweight BMI status, gender, and age as predictors for CRC, and Figure 2b presents the interaction profiles of four selected factors as examples of the interaction profiles. The lines of association with the CRC risk crossed and were non-parallel for the interaction between HEI and thiamine. Supplementary Figure S1a presents the profilers of these parameters with vegetable intake and the interaction profiles of the remaining parameters. The lines of association with CRC risk crossed and were non-parallel for overweight BMI status, with gender and BMI interacting with the *MTHFR* 677 polymorphism (see Supplementary Figure S1b) as gene-environment interactions.

### 3.5. Predictive Model

Using the most influential variables (Table 4), two GR models were developed using LOO cross validation methods to predict the probability of CRC. GR is also known as penalized regression. As the name implies, the modeling process penalizes complicated models to avoid overfitting. Hence, compared with conventional regression modeling methods, such as LR, GR tends to yield a more optimal model. In each case, the models were first compared to the conventional baseline LR model through validation. The parameter estimates along with the associated *p*-values for the baseline LR results with validation are shown in the left panel of Table 5 and Supplementary Table S5, including the parameter estimates for effect sizes and 95% confidence intervals (CI). Then, two GR models were developed using the Adaptive Elastic Net method with AICc validation and the Adaptive Elastic Net method with LOO cross validation to predict the probability of cancer (the middle and right panels of Table 5 and Supplementary Table S5).

In Supplementary Table S5, a seven-factor model with a baseline conventional LR model was constructed with two significant interactions—thiamine and HEI 77, and gender and overweight as measured by BMI status—And four significant individual parameters associated with these interactions and three additional individual factors: the total polymorphism score, age (median: 56), and vegetable intake (10 oz) (all *p* < 0.05 except vegetable intake: *p* < 0.1). While the effect of overweight status was not significant, it must be included in the models because of its interaction with gender. The GR LOO validation model was the best model with the lowest misclassification rate (0.22) and the highest AUC coverage (0.85, Supplementary Figure S2). In regard to significant parameters Both GR models presented the HEI (score of 77) and thiamine (100% RDI), and possibly vegetable intake (10 oz), as modifiable factors, in addition to the total polymorphisms of five genes in the OCM pathway and demographic characteristics of age and gender as predictors of cancer. While the total polymorphism score was a significant parameter for both GR models, it was not significant for the conventional LR model.

**Figure 3:**
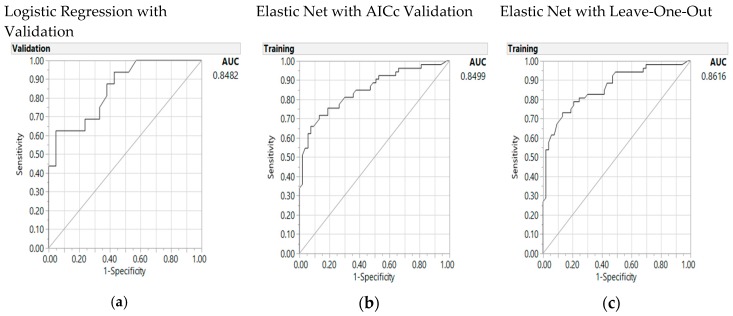
Area under the receiver operating characteristic curve (AUC) for the baseline logistic regression model (**a**), the Elastic Net with Akaike’s information criteria with correction (AICc) validation model (**b**), and the Leave-One-Out validation model (**c**) for the predictors of colorectal cancer with addition of the *MTHFR* 677 polymorphism and its interaction with overweight status.

When *MTHFR* 677 was added into the predictive model (Table 5) to give an eight-factor model, the same significant interaction terms were noted as associated factors. The misclassification rate for the Elastic Net LOO validation, shown in Table 5 on the right, was the lowest at 0.21, and the baseline LR (on the left) also presented a best and lower rate of 0.22, whereas the AICcs were similar to the earlier model, as shown in Table S5. The Elastic Net LOO validation outperformed the LR model with a lower misclassification rate, AUC, and the identification of more significant parameters, again leaving out overweight status due to its “0” parameter estimate and a *p* value of “1”. The AUCs (Figure 3) were 0.86 for the Elastic Net LOO model (right panel), and 0.85 for both the Elastic Net AICc validation model (middle panel) and the LR model (left panel). Vegetable intake (10 oz) was shown to be a significant parameter in the GR LOO model, whereas the interaction of *MTHFR* 677 with overweight BMI) was approaching significance with a *p* value of 0.059. Only four out of 11 parameters (three interactions and eight individual factors) including only one interaction term (HEI and thiamine) were significant in the LR models, compared to eight out of 11 tested parameters being significant in both GR models.

To illustrate the effects of different factors on these predictive models, Table S6 presents a series of models by progressively including the additional factors presented in the Table 5. The *p*-values for the significance of the parameter estimates, misclassification rates, AICc, and AUCs of the individual variables (i.e., HEI, thiamine, overweight BMI status, gender, total gene polymorphism mutation score, age, vegetable intake, *MTHFR* 677, and total folate intake) and their significant interactions were included in these illustrative progressions. As shown in Table S6, the misclassification rate was the lowest and best in the models presented in Table 5, the GR LOO model (0.21 versus 0.24 in one more factor or one less factor models) and the AICcs in the GR AICc validation, compared to the other GR models tested. Adding folate intake as an additional parameter to give a nine-factor model increased the misclassification rates for the LR and GR LOO models, while the inclusion of folate as a parameter did not reach significance (see Supplementary Table S6).

## 4. Discussion

Using supervised machine-learning analytics, we presented a ground-breaking predictive modeling study which gives improved prediction accuracy and the best fitted model, to identify significant predictors including interaction terms. We found the significant predictors of CRC and built prediction models using identified predictors of importance. We observed a composite of five key genes in the OCM pathway; the dietary parameters of thiamine and a HEI score of 77 and their interactions; and age, gender, and overweight status and their interactions as predictors of cancer in multiethnic CRC families. In addition, through the dimension reduction approach, which recognizes the variables of importance, the best predictive model was generated using the GR models, Elastic Net AICc validation and LOO cross validation methods. We observed the HEI as modifiable dietary factor and OCM related genetic factors as independent factors for CRC risk in this study. In addition to the HEI, other significant dietary predictors found in this study included thiamine and vegetable intake, which are converging dietary risk factors for CRC, to demonstrate that the findings related to the HEI dietary parameters presented as a composite score were not due to chance. Additionally, the prediction models presented in this study were better than conventional models presented in previous studies at identifying potential interactive parameters, addressing improved accuracy (lower misclassification rate and AUC), and recognizing the fitness of models with AICc. No previous studies have validated their predictions with added criteria to achieve rigor and reproducibility in their results. While aging and demographic characteristics such as gender might not be modifiable in the prevention of cancer, it is promising to see that dietary parameters play significant roles in the cancer prediction (as shown through the supervised machine learning based GR models with validations). Healthy eating as a modifiable habit is particularly promising due to its beneficial intervention against mutated genes in the OCM pathways which place a patient at a higher risk of cancer. 

HEI interacted with thiamine (Vitamin B1), which is a new finding for the literature. Thiamine is tested as part of the RDI analysis, and the natural food sources for thiamine include wholegrains, legumes, and some meats and fish which HEI scoring included as part of a healthy diet (versus limiting portions of salt, saturated fat and empty calories). Both gender and the *MTHFR* 677 polymorphism interacted with overweight BMI status in the prediction of CRC, with the cancer group having more men and more overweight cases. While previous studies tested the association of higher HEI scores with lower CRC risk [47,48,49,50,51,52], we further documented the scale of HEI with a median split distribution (a score of 77 versus 80) for the best predictive model in predicting the CRC risk with the diverse sample used in this study. The HEI score was significantly split at the median score of 77 into greater or less scores, confirmed through the machine-learning recursive tree method and the predictive modeling, while an HEI score of greater than 80 is the set value for a good diet according to the US national standard [44,45,46]. The results showed that the HEI and healthy eating are modifiable factors for healthy living, in addition to the genes in the OCM pathway. Personalized nutrition can be planned when patients present increased gene mutations in the OCM pathway, particularly by having heightened awareness of supplying methyl donors to improve health outcomes. 

CRC is a disease that comprises a group of molecularly heterogeneous diseases that are characterized by a range of genomic and epigenomic alterations [38]. Therefore, genes, diet, and interactive parameters may increase the risk of CRC due to specific molecular features. For example, a recent study demonstrated an association between pro-inflammatory diets, such as those including red and processed meats, refined grains, and carbonated drinks, and a higher risk for CRC subtypes with absent/low-lymphocytic reactions than CRC subtypes with high-lymphocytic reactions in the tumor microenvironment. The pro-inflammatory diet-associated CRC subtype was shown to be hypermutated CRC with microsatellite instability (MSI), the CpG (cytosine and guanine separated by only one phosphate group) island methylation phenotype (CIMP), and the *BRAF* wild-type phenotype [38,39]. While previous studies presented gene–environment interactions, associating genes in the OCM pathway [73,74,77] related to CRC prevention [73,77], we applied new GR predictive modeling and validation analytics methods using JMP pro programming (SAS Institute, Cary, NC, USA). We used the supervised machine-learning based analytics with the target variable being specified as cancer status and included the ensemble methods and the GR Elastic Net methods that are well-known remedies for small-sample studies to validate the analyses using random subsets of samples [96] in the best fit models. These analytics presented converging parameters for the reproducibility and rigor of the predictive modeling. While some family participants in this study shared genetic heritage with the cancer cases, the CRC group had increased combined gene mutations in the OCM pathway than the control group in this family-based study. The finding that healthy eating is a modifiable factor for cancer prevention is promising and encouraging to the families with CRC history. 

Our sample size was limited with a total of 106 participants: 53 CRC cases and 53 matched family/friend controls. For the predictive modeling construction using the GR Elastic Net LOO model, we did not have a sufficient number of samples from any of the four racial–ethnic subgroups to generate stable results for the racial ethnic subgroups. Elastic models and machine learning techniques (classification tree/bootstrap random forest) are designated to build a parsimonious predictive model by selecting variables of importance or applying shrinkage penalties to variables of less significance. For small sample sizes, as in this article, they should serve the intended purpose. Data-driven selection approaches like LASSO or random forest are not stochastic, a factor that conventional model inference requires for its sampling distribution. While elastic models like LASSO could provide estimates with less variance, they may also introduce a certain degree of bias into the parameter estimates [98]. For valid parameter estimation in our small dataset, we included bootstrapping and conventional LR with parameter estimates for effect sizes and confidence intervals, as recommended previously [98]. The Elastic Net method is suitable for handling data sets with many variables and few observations. In the Elastic Net method, a stage-wise algorithm called LARS-EN [90,91] efficiently finds the best solution path and it is more likely to balance variance and bias than other methods. In summary, future studies with larger samples are needed to generate stable results and to further validate these findings for various racial-ethnic groups. Caution is warranted when interpreting the results of this study for various ethnic groups, as there is potential for inflated Type I errors due to multiple testing of the models and not adjusting *p*-values for the small sample sizes. Further studies involving gene-environment/diet interactions using larger diverse samples should be designed to validate these findings. 

In summary, we examined genetic, demographic, and dietary parameters and related interactions in preparation for the precision-based healthcare era for cancer prevention and to improve health outcomes for personalized nutrition. We used a cross-validation approach to predict the risk of CRC from individual parameters and related interactions in relation to OCM and inflammatory pathways. For family-centered healthcare, the family-based design can provide further evidence on the most efficient and effective interventions to prevent cancer, as family members can help to provide more accurate monitoring and sustained eating habits [56,97]. Future studies may focus on the epigenetics of methyl donors from healthy eating related to folate metabolism and its mechanisms to achieve healthy living and cancer prevention.

## Figures and Tables

**Table 1:** Comparisons of demographic/environmental factors between the control and cancer groups.

Parameters		Control (*N* = 53) *n* (%)	Cancer (*N* = 53) *n* (%)	*p*
Gender	Male	14 (26%)	25 (47%)	0.027
	Female	39 (74%)	28 (53%)	
Age (years)	Mean ± SD	47 ± 17	61 ± 11	<0.0001
	Range	18–80	37–79	
Ethnicity	Asian	22 (42%)	18 (34%)	0.88
	Caucasian	16 (30%)	18 (34%)	
	Hispanic	11 (21%)	12 (23%)	
	African American	4 (7.5%)	5 (9.4%)	
BMI status	Obese	11 (21%)	15 (28%)	0.37
Alcohol drinker	Yes	25 (47%)	32 (60%)	0.17
Smoker	Yes	5 (9.4%)	4 (7.6%)	0.73
Total polymorphisms (0–6)	≥4	16 (30%)	27 (51%)	0.03

BMI: body mass index. SD: standard deviation.

**Table 2:** Comparisons of dietary parameters in the Healthy Eating Index between the control and cancer groups.

Parameters (Amount, Score)	Control (*N* = 53) Mean ± SD	Cancer (*N* = 53) Mean ± SD	*p*
Calorie (per day)	1640 ± 1021	1603 ± 784	0.84
Total Fruit (≥0.8 cup, 5 points)	1.6 ± 1.5	1.6 ± 1.4	0.98
Whole Fruit (≥0.4 cup, 5 points)	1.2 ± 1.1	1.2 ± 1.0	0.95
Vegetables (≥1.1 cups, 5 points)	1.6 ± 1.1	1.5 ± 1.3	0.86
Dark Green (≥0.4 cup, 5 points)	0.9 ± 0.7	0.8 ± 0.7	0.66
Total Grains (≥3 oz, 5 points)	4.6 ± 3.3	4.6 ± 2.7	0.95
Whole Grains (≥1.5 oz, 5 points)	1.5 ± 1.4	2.0 ± 1.9	0.16
Dairy (≥1.3 cups, 10 points)	1.8 ± 4.3	1.0 ± 1.2	0.19
Protein (≥2.5 oz, 10 points)	6.3 ± 5.0	5.3 ± 3.2	0.22
Oil and Nuts (≥12 g. 10 points)	37 ± 25	36 ± 19	0.72
Saturated Fat (g, ≤8% energy)	18 ± 9.6	19 ± 13	0.82
Sodium (≤1.1 g. 10 points)	3.3 ± 2.1	3.0 ± 1.8	0.34
Empty Calories (≤19% energy)	348 ± 235	353 ± 216	0.91
HEI score (≤50, 51–79, ≥80)	75 ± 10	76 ± 8.3	0.43
HEI score (≥77)	24 (45%)	30 (57%)	0.24
HEI score (≥80)	20 (38%)	21 (40%)	0.84

SD: standard deviation, oz: ounce, HEI: Healthy Eating Index.

**Table 3:** Comparisons of recommended daily intakes between control and cancer groups.

Parameters, Unit, RDI		Control (*N* = 53) *n* (%)	Cancer (*N* = 53) *n* (%)	*p*
Carbohydrates, g, 45–65% calorie	≥45%	38 (73%)	37 (70%)	0.83
Protein, g, 10–35% calorie	≥20%	21 (40%)	17 (32%)	0.42
Total Fat, g, 20–35% calorie	<35%	35 (66%)	34 (64%)	0.84
Saturated Fat, g, <10% calorie	<10%	28 (53%)	23 (43%)	0.33
Cholesterol, <300 mg	<100%	39 (74%)	39 (74%)	1.00
Sodium, <2300 mg	<100%	19 (36%)	21 (40%)	0.69
Fiber, ≥25 g	≥100%	9 (17%)	7 (13%)	0.59
Total Folate, 400 mcg	≥100%	13 (25%)	21 (40%)	0.10
Vitamin B1 (Thiamine), 1.1 mg	≥100%	30 (57%)	35 (66%)	0.32
Vitamin B2 (Riboflavin), 1.1 mg	≥100%	37 (70%)	41 (77%)	0.38
Vitamin B6, 1.3 mg	≥100%	35 (66%)	33 (62%)	0.69
Vitamin B12, 2.4 mcg	<150%	25 (47%)	19 (36%)	0.24
Niacin, 14 mg	≥100%	35 (66%)	37 (70%)	0.68
Calcium, 1000 mg	≥75%	24 (45%)	22 (42%)	0.70
Magnesium, 320 mg	≥75%	27 (51%)	25 (47%)	0.70
Iron, 8 mg	≥100%	19 (36%)	25 (47%)	0.24
Zinc, 8 mg	≥100%	27 (51%)	26 (49%)	0.85
Methionine, 13 mg/kg	<150%	22 (42%)	23 (43%)	0.84

RDI: recommended daily intake.

**Table 4:** Major dietary parameters as predictors of colorectal cancer.

Term	Number of Splits	*G* ^2^	Column Contribution	Portion
Age (≤ or >56 years)	61	3.12	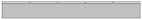	0.28
Gender	44	1.35	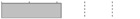	0.12
Total Polymorphisms (≥4)	49	1.30	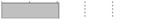	0.11
Total Vegetable Intake 10 oz	43	1.24	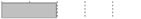	0.11
Total Folate Intake 100%	49	1.05	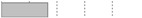	0.09
HEI 77	42	0.72	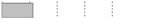	0.06
Overweight BMI	44	0.70	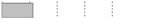	0.06
Vitamin B12 150%	35	0.66	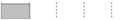	0.06
Thiamine 100%	38	0.65	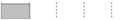	0.06
*MTHFR* 677 Polymorphism	39	0.52	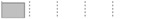	0.05

HEI: Health Eating Index.

**Table 5:** Gene-diet interactions including *MTHFR* 677 on the predictors of colorectal cancer: baseline logistic regression model and generalized regression Elastic Net models.

Parameters	Logistic Regression with Validation		Generalized Regression Elastic Net Model				
			AICc Validation		Leave-One-Out Validation		
	Estimate (95% CI)	*p* (*X*^2^)	Estimate (95% CI)	*p* (*X*^2^)		Estimate (95% CI)	*p* (*X*^2^)
(Intercept)	−0.59 (−2.5, 1.3)	0.54	−0.56 (−2.2, 1.1)	0.51		−1.02 (−2.5, 0.49)	0.19
Thiamine * HEI	−3.67 (−6.6, −0.79)	0.01	−2.80 (−5.1, −0.51)	0.02		−2.73 (−4.9, −0.56)	0.01
Gender * BMI Overweight	−2.4 (−5.1, 0.15)	0.06	−3.49 (−5.6, −1.4)	0.001		−3.36 (−5.2, −1.5)	0.0003
Gender	1.86 (0.07, 3.6)	0.04	2.50 (1.1, 3.9)	0.0005		2.53 (1.3, 3.8)	<0.0001
Total Polymorphisms	−0.95 (−2.2, 0.33)	0.15	−1.54 (−2.7, −0.35)	0.011		−1.65 (−2.8, −0.53)	0.004
HEI	2.73 (0.41, 5.1)	0.02	2.53 (0.35, 4.7)	0.02		2.52 (0.49, 4.6)	0.02
Thiamine	1.75 (−0.08, 3.6)	0.06	1.71 (0.18, 3.2)	0.03		1.86 (0.42, 3.3)	0.011
Age	−1.32 (−2.6, −0.08)	0.04	−1.48 (−2.5, −0.51)	0.003		−1.35 (−2.3, −0.41)	0.005
Vegetable 10 oz	1.20 (−0.19, 2.6)	0.09	1.03 (−0.07, 2.1)	0.07		1.02 (0.03, 2.0)	0.04
*MTHFR 677* * BMI	1.42 (−1.2, 4.0)	0.29	2.02 (−0.07, 4.1)	0.06		1.43 (−0.29, 3.2)	0.10
*MTHFR 677*	−0.63 (−2.4, 1.1)	0.48	0.63 (−1.9, 0.63)	0.33		−0.14 (−1.3, 1.1)	0.82
BMI Overweight	−0.36 (−2.3, 1.5)	0.71	−0.33 (−1.9, 1.2)	0.68		0 (0, 0)	1.00
Misclassification Rate	0.22		0.25			0.21	
AICc	71		130			n/a	
Area Under the Curve	0.85		0.85			0.86	

CI: confidence interval; *: Interaction terms, HEI: Health Eating Index score, AICc: Akaike’s information criterion with correction, n/a: not available.

## Supplementary Materials

The following are available online at http://www.mdpi.com/2072-6643/10/6/795/s1: Table S1. Comparisons on demographic/environmental factors per race groups; Table S2. Comparisons on dietary parameters: Healthy Eating Index per race groups; Table S3. Comparisons on dietary parameters: Recommended daily intakes per race groups; Table S4. Major dietary parameters as predictors of colorectal cancer; Table S5. Gene-diet interactions on the predictors of colorectal cancer: Baseline logistic regression model and generalized regression Elastic Net models; Table S6. Baseline logistic regression and generalized regression Elastic Net on the selected predictors of colorectal cancer from genetic and dietary factors; Figure S1. Gene-diet Interaction: (a). prediction profiler, interaction profiles – gender interacting with overweight per body mass index (BMI), MTHFR 677 polymorphism interacting with overweight based on BMI; Figure S2. Area under the receiver operating characteristic curve (AUC) for baseline logistic regression model (left panel), Elastic Net with Akaike’s information criteria with correction (AICc) validation model (middle) and Leave-One-Out validation model (right panel) on the predictors of colorectal cancer; Supplementary File: Consent Form (Template).

## References

[1] Magalhães B., Peleteiro B., Lunet N. (2012). Dietary patterns and colorectal cancer: Systematic review and meta-analysis. Eur. J. Cancer Prev..

[2] Fan Y., Jin X., Man C., Gao Z., Wang X. (2017). Meta-analysis of the association between the inflammatory potential of diet and colorectal cancer risk. Oncotarget.

[3] Tárraga López P.J., Albero J.S., Rodríguez-Montes J.A. (2014). Primary and secondary prevention of colorectal cancer. Clin. Med. Insights Gastroenterol..

[4] Cavicchia P.P., Steck S.E., Hurley T.G., Hussey J.R., Ma Y., Ockene I.S., Hebert J.R. (2009). A new dietary inflammatory index predicts interval changes in serum high-sensitivity C.-reactive protein. J. Nutr..

[5] Shivappa N., Steck S.E., Hurley T.G., Hussey J.R., Hebert J.R. (2014). Designing and developing a literature-derived, population-based dietary inflammatory index. Public. Health Nutr..

[6] Johnson C.M., Wei C., Ensor J.E., Smolenski D.J., Amos C.I., Levin B., Berry D.A. (2013). Meta-analyses of colorectal cancer risk factors. Cancer Causes Control.

[7] WCRF-AICR Continuous Update Project Diet, Nutrition, Physical Activity and Colorectal Cancer. 2017. World Cancer Research Fund International/American Institute for Cancer Research. http://www.aicr.org/continuous-update-project/reports/colorectal-cancer-2017-report.pdf.

[8] Holden D.J., Harris R., Porterfield D.S., Jones D.E., Morgan L.C., Reuland D., Gilehrist M., Viswanathan M., Lohr K.N., Lynda-MdDonald B. (2010). Enhancing the use of quality of colorectal cancer screening. Evid. Rep. Technol. Assess..

[9] Visser A., Vrieling A., Murugesu L., Hoogerbrugge N., Kampman E., Hoedjes M. (2017). Determinants of adherence to recommendations for cancer prevention among Lynch Syndrome mutation carriers: A qualitative exploration. PLoS ONE.

[10] Campbell P.T., Curtin K., Ulrich C.M., Samowitz W.S., Bigler J., Velicer C.M., Caan B., Potter J.D., Slattery M.L. (2009). Mismatch repair polymorphisms and risk of colon cancer, tumour microsatellite instability and interactions with lifestyle factors. Gut.

[11] Bishehsari F., Mahdavinia M., Vacca M., Malekzadeh R., Mariani-Costantini R. (2014). Epidemiological transition of colorectal cancer in developing countries: Environmental factors, molecular pathways, and opportunities for prevention. World J. Gastroenterol..

[12] Shiao S.P., Yu C.H. (2016). Meta-Prediction of MTHFR Gene polymorphism mutations and associated risk for colorectal cancer. Biol. Res. Nurs..

[13] Wu S.-M., Chen Z.-F., Young L., Shiao S.P.K. (2017). Meta-prediction of the effects of Methylenetetrahydrofolate reductase gene polymorphisms and air pollution on risk of Alzheimer’s disease. Int. J. Environ. Res. Public Health.

[14] Lien S.-Y.A., Young L., Gau B.-S., Shiao S.P.K. (2017). Meta-prediction of MTHFR gene polymorphism-mutations, air pollution, and risks of leukemia among world populations. Oncotarget.

[15] Gonzales M.C., Yu P.-J., Shiao S.P.K. (2017). Meta-prediction of MTHFR gene polymorphism-mutations and air pollution as risk factors for breast cancer. Nurs. Res..

[16] Yang Y.L., Yang H.L., Shiao S.P.K. (2018). Meta-prediction of MTHFR gene polymorphisms and air pollution on the risk of hypertensive disorders in pregnancy worldwide. Int. J. Environ. Res. Public Health.

[17] Cadet J., Douki T., Ravanat J.L. (2010). Oxidatively generated base damage to cellular DNA. Free Radic. Biol. Med..

[18] Hair J.M., Terzoudi G.I., Hatzi V.I., Lehockey K.A., Srivastava D., Wang W., Pantelias G.E., Georgakilas A.G. (2010). BRCA1 role in the mitigation of radiotoxicity and chromosomal instability through repair of clustered DNA lesions. Chem. Biol. Interact..

[19] Baccarelli A., Cassano P.A., Litonjua A., Park S.K., Suh H., Sparrow D., Vokonas P., Schwartz J. (2008). Cardiac autonomic dysfunction: Effects from particulate air pollution and protection by dietary methyl nutrients and metabolic polymorphisms. Circulation.

[20] Taioli E., Garza M.A., Ahn Y.O., Bishop D.T., Bost J., Budai B., Chen K., Gemignani F., Keku T., Lima C.S. (2009). Meta- and pooled analyses of the methylenetetrahydrofolate reductase (MTHFR) C677T polymorphism and colorectal cancer: A HuGE-GSEC review. Am. J. Epidemiol..

[21] Kennedy D.A., Stern S.J., Matok I., Moretti M.E., Sarkar M., Adams-Webber T., Koren G. (2012). Folate intake, MTHFR polymorphisms, and the risk of colorectal cancer: A systematic review and meta-analysis. J. Cancer Epidemiol..

[22] Zhang D., Wen X., Wu W., Guo Y., Cui W. (2015). Elevated Homocysteine Level and Folate Deficiency Associated with Increased Overall Risk of Carcinogenesis: Meta-Analysis of 83 Case-Control Studies Involving 35,758 Individuals. PLoS ONE.

[23] Anderson O.S., Sant K.E., Dolinoy D.C. (2012). Nutrition and epigenetics: An interplay of dietary methyl donors, one-carbon metabolism and DNA methylation. J. Nutr. Biochem..

[24] Ryan-Harshman M., Aldoori W. (2007). Diet and colorectal cancer: Review of the evidence. Can. Fam. Phys..

[25] Turati F., Bravi F., Di Maso M., Bosetti C., Polesel J., Serraino D., Dalmartello M., Giacosa A., Montella M., Tavani A. (2017). Adherence to the World Cancer Research Fund/American Institute for Cancer Research recommendations and colorectal cancer risk. Eur. J. Cancer.

[26] Tabung F.K., Brown L.S., Fung T.T. (2017). Dietary Patterns and Colorectal Cancer Risk: A Review of 17 Years of Evidence (2000–2016). Curr. Colorectal Cancer Rep..

[27] Vieira A.R., Abar L., Chan D.S.M., Vingeliene S., Polemiti E., Stevens C., Greenwood D., Norat T. (2017). Foods and beverages and colorectal cancer risk: A systematic review and meta-analysis of cohort studies, an update of the evidence of the WCRF-AICR Continuous Update Project. Ann. Oncol..

[28] Klai S., Fekih-Mrissa N., El Housaini S., Kaabechi N., Nsiri B., Rachdi R., Gritli N. (2011). Association of MTHFR A1298C polymorphism (but not of MTHFR C677T) with elevated homocysteine levels and placental vasculopathies. Blood Coagul. Fibrinolysis.

[29] Yang B., Liu Y., Li Y., Fan S., Zhi X., Lu X., Wang D., Zheng Q., Wang Y., Wang Y. (2013). Geographical distribution of MTHFR C677T, A1298C and MTRR A66G gene polymorphisms in China: Findings from 15357 adults of Han nationality. PLoS ONE.

[30] Frosst P., Blom H.J., Milos R., Goyette P., Sheppard C.A., Matthews R.G., Boers G.J., den Heijer M., Kluijtmans L.A., van den Heuvel L.P. (1995). A candidate genetic risk factor for vascular disease: A common mutation in methylenetetrahydrofolate reductase. Nat. Genet..

[31] Yaliwal L.V., Desai R.M. (2012). Methylenetetrahydrofolate reductase mutations, a genetic cause for familial recurrent neural tube defects. Indian J. Hum. Genet..

[32] Ravegnini G., Zolezzi Moraga J.M., Maffei F., Musti M., Zenesini C., Simeon V., Sammarini G., Festi D., Hrelia P., Angelini S. (2015). Simultaneous analysis of SEPT9 promoter methylation status, micronuclei frequency, and folate-related gene polymorphisms: The potential for a novel blood-based colorectal cancer biomarker. Int. J. Mol. Sci..

[33] Selhub J., Rosenberg I.H. (2016). Excessive folic acid intake and relation to adverse health outcome. Biochimie.

[34] Cheng T.Y., Makar K.W., Neuhouser M.L., Miller J.W., Song X., Brown E.C., Beresford S.A., Zheng Y., Poole E.M., Galbraith R.L. (2015). Folate-mediated one-carbon metabolism genes and interactions with nutritional factors on colorectal cancer risk: Women’s Health Initiative Observational Study. Cancer.

[35] Li W.X., Dai S.X., Zheng J.J., Liu J.Q., Huang J.F. (2015). Homocysteine metabolism gene polymorphisms (MTHFR C677T, MTHFR A1298C, MTR A2756G and MTRR A66G) jointly elevate the risk of folate deficiency. Nutrients.

[36] Lucock M., Yates Z., Martin C., Choi J.H., Beckett E., Boyd L., LeGras K., Ng X., Skinner V., Wai R. (2015). Methylation diet and methyl group genetics in risk for adenomatous polyp occurrence. BBA Clin..

[37] Zhou D., Mei Q., Luo H., Tang B., Yu P. (2012). The polymorphisms in methylenetetrahydrofolate reductase, methionine synthase, methionine synthase reductase, and the risk of colorectal cancer. Int. J. Biol. Sci..

[38] Inamura K. (2018). Colorectal Cancers: An Update on Their Molecular Pathology. Cancers.

[39] Liu L., Nishihara R., Qian Z.R., Tabung F.K., Nevo D., Zhang X., Song M., Cao Y., Mima K., Masugi Y. (2017). Association Between Inflammatory Diet Pattern and Risk of Colorectal Carcinoma Subtypes Classified by Immune Responses to Tumor. Gastroenterology.

[40] How AICR Recommendations Cuts Colorectal Cancer Risk for Both Men and Women. http://www.aicr.org/cancer-research-update/2016/11_02/cru-how-AICR-recommendations-cuts-colorectal-cancer-risk-for-men-and-women.html.

[41] Hastert T.A., White E. (2016). Association between meeting the WCRF/AICR cancer prevention recommendations and colorectal cancer incidence: Results from the VITAL cohort. Cancer Causes Control.

[42] Yuan Y.-Q., Li F., Dong R.-H., Chen J.-S., He G.-S., Li S.G., Chen B. (2017). The Development of a Chinese Healthy Eating Index and Its Application in the General Population. Nutrients.

[43] United States (U.S.) Department of Health and Human Services and U.S. Department of Agriculture (2015). 2015–2020 Dietary Guidelines for Americans. http://health.gov/dietaryguidelines/2015/guidelines/.

[44] United States Department of Agriculture (USDA) (2016). Healthy Eating Index (HEI). https://www.cnpp.usda.gov/healthyeatingindex.

[45] National Institute of Health (NIH) Nutrient Recommendations: Dietary Reference Intakes (DRI). (n.d.). https://ods.od.nih.gov/Health_Information/Dietary_Reference_Intakes.aspx.

[46] Panizza C.E., Shvetsov Y.B., Harmon B.E., Wilkens L.R., Le Marchand L., Haiman C., Reedy J., Boushey C.J. (2018). Testing the Predictive Validity of the Healthy Eating Index-2015 in the Multiethnic Cohort: Is the Score Associated with a Reduced Risk of All-Cause and Cause-Specific Mortality?. Nutrients.

[47] Djuric Z., Severson R.K., Kato I. (2012). Association of dietary quercetin with reduced risk of proximal colon cancer. Nutr. Cancer.

[48] Miller P.E., Lazarus P., Lesko S.M., Muscat J.E., Harper G., Cross A.J., Sinha R., Ryczak K., Escobar G., Mauger D.T. (2010). Diet index-based and empirically derived dietary patterns are associated with colorectal cancer risk. J. Nutr..

[49] Reedy J., Wirfält E., Flood A., Mitrou P.N., Krebs-Smith S.M., Kipnis V., Midthune D., Leitzmann M., Hollenbeck A., Schatzkin A. (2010). Comparing 3 dietary pattern methods—Cluster analysis, factor analysis, and index analysis—With colorectal cancer risk: The NIH-AARP Diet and Health Study. Am. J. Epidemiol..

[50] Jiang Q., Chen K., Ma X., Li Q., Yu W., Shu G., Yao K. (2005). Diets, polymorphisms of Methylenetetrahydro- folate reductase, and the susceptibility of colon cancer and rectal cancer. Cancer Detect. Prev..

[51] Guerreiro C.S., Cravo M.L., Brito M., Vidal P.M., Fidalgo P.O., Leitão C.N. (2007). The D1822V APC polymorphism interacts with fat, calcium, and fiber intakes in modulating the risk of colorectal cancer in Portuguese persons. Am. J. Clin. Nutr..

[52] May-Wilson S., Sud A., Law P.J., Palin K., Tuupanen S., Gylfe A., Hänninen U.A., Cajuso T., Tanskanen T., Kondelin J. (2017). Pro-inflammatory fatty acid profile and colorectal cancer risk: A Mendelian randomization analysis. Eur. J. Cancer.

[53] De Vogel S., Wouters K.A., Gottschalk R.W., van Schooten F.J., de Goeij A.F., de Bruïne A.P., Goldbohm R.A., van den Brandt P.A., van Engeland M., Weijenberg M.P. (2011). Dietary methyl donors, methyl metabolizing enzymes, and epigenetic regulators: Diet-gene interactions and promoter CpG island hypermethylation in colorectal cancer. Cancer Causes Control.

[54] Sharp L., Little J., Brockton N.T., Cotton S.C., Masson L.F., Haites N.E., Cassidy J. (2008). Polymorphisms in the methylenetetrahydrofolate reductase (MTHFR) gene, intakes of folate and related B vitamins and colorectal cancer: A case-control study in a population with relatively low folate intake. Br. J. Nutr..

[55] Shiao S.P.K., Grayson J., Yu C.H., Wasek B., Bottiglieri T. (2018). Gene Environment Interactions and Predictors of Colorectal Cancer in Family-Based, Multi-Ethnic Groups. J. Pers. Med..

[56] Simidjievski N., Todorovski L., Džeroski S. (2016). Modeling dynamic systems with efficient ensembles of process-based models. PLoS ONE.

[57] Khalilia M., Chakraborty S., Popescu M. (2011). Predicting disease risks from highly imbalanced data using random forest. BMC Med. Inform. Decis. Mak..

[58] Islam M.M., Yao X., Shahriar Nirjon S.M., Islam M.A., Murase K. (2008). Bagging and boosting negatively correlated neural networks. IEEE Trans. Syst. Man Cybern. B Cybern..

[59] Wang C.W. (2006). New ensemble machine learning method for classification and prediction on gene expression data. Conf. Proc. IEEE Eng. Med. Biol. Soc..

[60] Friedman J., Hastie T., Tibshirani R. (2010). Regularization paths for generalized linear models via coordinate descent. J. Stat. Softw..

[61] Song L., Langfelder P., Horvath S. (2013). Random generalized linear model: A highly accurate and interpretable ensemble predictor. BMC Bioinform..

[62] Witten D.M., Tibshirani R. (2009). Covariance-regularized regression and classification for high-dimensional problems. J. R. Stat. Soc. Ser. B Stat. Methodol..

[63] Wu Y. (2012). Elastic Net for Cox’s proportional hazards model with a solution path algorithm. Stat. Sin..

[64] Krist A.H., Glenn B.A., Glasgow R.E., Balasubramanian B.A., Chambers D.A., Fernandez M.E., Heurtin-Roberts S., Kessler R., Ory M.G., Phillips S.M. (2013). Designing a valid randomized pragmatic primary care implementation trial: The my own health report (MOHR) project. Implement. Sci..

[65] Center for Disease Control and Prevention (CDC). 2012. National Health and Nutrition Examination Survey. Center for Disease Control and Prevention. http://www.cdc.gov/nchs/nhanes/nhanes_questionnaires.htm.

[66] National Coalition for Health Professional Education in Genetics Family History Educational Aids. http://www.nchpeg.org/index.php?option=com_content&view=article&id=145&Itemid=64.

[67] Lievers K.J., Boers G.H., Verhoef P., Heijer M., Kluijtmans L.A., Put N.M., Trijbels F.J., Blom H.J. (2001). A second common variant in the methylenetetrahydrofolate reductase (MTHFR) gene and its relationship to MTHFR enzyme activity, homocysteine, and cardiovascular disease risk. J. Mol. Med..

[68] Wren M.E., Shirtcliff E.A., Drury S.S. (2015). Not all biofluids are created equal: Chewing over salivary diagnostics and the epigenome. Clin. Ther..

[69] Torres-Sánchez L., Chen J., Díaz-Sánchez Y., Palomeque C., Bottiglieri T., López-Cervantes M., López-Carrillo L. (2006). Dietary and genetic determinants of homocysteine levels among Mexican women of reproductive age. Eur. J. Clin. Nutr..

[70] Neuhouser M.L., Kristal A.R., McLerran D., Patterson R.E., Atkinson J. (1999). Validity of short food frequency questionnaires used in cancer chemoprevention trials: Results from the Prostate Cancer Prevention Trial. Cancer Epidemiol. Biomark. Prev..

[71] Patterson R.E., Kristal A.R., Tinker L.F., Carter R.A., Bolton M.P., Agurs-Collins T. (1999). Measurement characteristics of the Women’s Health Initiative food frequency questionnaire. Ann. Epidemiol..

[72] Schakel S.F., Sievert Y.A., Buzzard I.M. (1988). Sources of data for developing and maintaining a nutrient database. J. Am. Diet. Assoc..

[73] Harnack L., Lee S., Schakel S.F., Duval S., Luepker R.V., Arnett D.K. (2003). Trends in the trans-fatty acid composition of the diet in a metropolitan area: The Minnesota Heart Survey. J. Am. Diet Assoc..

[74] Young L., Shiao S.P.K. Validation of Methyl Donors between Two Food Measurements in a Colorectal Cancer Study. http://www.aacr.org/Documents/AACR2017_Proceedings.pdf.

[75] Health Information Nutrient recommendations: Dietary reference intakes. U. S. Department of Health and Human Services, National Institutes of Health, Office of Dietary Supplements. https://ods.od.nih.gov/Health_Information/Dietary_Reference_Intakes.aspx.

[76] Zhao L.P., Le Marchand L. (1992). An analytical method for assessing patterns of familial aggregation in case-control studies. Genet. Epidemiol..

[77] Grayson J., Gardner S., Stephens M. (2015). Building Better Models with JMP^®^ Pro. 2015.

[78] Klimberg R., McCullough B.D. (2016). Fundamentals of Predictive Analytics with JMP.

[79] Yu C.H., Osborne J. (2007). Resampling: A Conceptual and Procedural Introduction. Best Practices in Quantitative Methods.

[80] Faraway J.J. (2005). Extending the Linear Model with R: Generalized Linear, Mixed Effects and Nonparametric Regression Models (Texts in Statistical Science).

[81] Meir R., Rätsch G., Mendelson S., Smola A.J. (2003). An Introduction to Boosting and Leveraging. Advanced Lectures on Machine Learning. Lecture Notes in Computer Science.

[82] Zaman M.F., Hirose H. (2011). Classification performance of bagging and boosting type ensemble methods with small training sets. New Gener. Comput..

[83] Burnham K.P., Anderson D.R. (2002). Model Selection and Multimodel Inference: A Practical Information-Theoretic Approach.

[84] Burnham K.P., Anderson D.R. (2004). Multimodel inference: Understanding AIC and BIC in model selection. Sociol. Meth. Res..

[85] Yang Y. (2005). Can the strengths of AIC and BIC be shared?. Biometrika.

[86] Akaike H. (1974). A new look at the statistical model identification. IEEE Trans. Autom. Control.

[87] Akaike H. (1978). A Bayesian analysis of the minimum AIC procedure. Ann. Inst. Stat. Math..

[88] SAS Institute Inc (2016). JMP 13 Fitting Linear Models.

[89] Cheng H., Garrick D.J., Fernando R.L. (2017). Efficient strategies for leave-one-out cross validation for genomic best linear unbiased prediction. J. Anim. Sci. Biotechnol..

[90] Zou H., Hastie T. (2005). Regularization and variable selection via the elastic net. J. R. Stat. Soc. B.

[91] Efron B., Hastie T., Johnstone I., Tibshirani R. (2004). Least angle regression. Ann. Stat..

[92] SAS Institute (2017). Overview of the Generalized Regression Personality.

[93] Crotty M., Barker C. (2014). Penalizing Your Models: An Overview of the Generalized Regression Platform.

[94] Tibshirani R. (2011). Regression shrinkage and selection via the lasso: A retrospective. J. R. Stat. Soc. Ser. B Stat. Methodol..

[95] Gonzales G.B., de Saeger S. (2018). Elastic net regularized regression for time-series analysis of plasma metabolome stability under sub-optimal freezing condition. Sci. Rep..

[96] Shmueli G. (2010). To Explain or to Predict?. Stat. Sci..

[97] Diaconis P., Efron B. (1983). Computer-intensive methods in statistics. Sci. Am..

[98] Cenit M.C., Olivares M., Codoñer-Franch P., Sanz Y. (2015). Intestinal microbiota and celiac disease: Cause, consequence or co-evolution?. Nutrients.

[99] Lu S., Liu Y., Yin L., Zhang K. (2017). Confidence intervals and regions for the lasso by using stochastic variational inequality techniques in optimization. J. R. Stat. Soc..

