# Supplementary material for: Personalized Nutrition—Genes, Diet, and Related Interactive Parameters as Predictors of Cancer in Multiethnic Colorectal Cancer Families
Source: Nutrients. 2018 Jun 20;10(6):795. doi: 10.3390/nu10060795 (PMC6024706; doi:10.3390/nu10060795)
Supplement: Supplementary file 1 [file nutrients-10-00795-s001.pdf]

Supplementary File: Personalized Nutrition—Genes, Diet, and Related Interactive Parameters as Predictors of Cancer in Multiethnic Colorectal Cancer Families

S. Pamela K. Shiao*, James Grayson, Amanda Lie, Chong Ho Yu; *Corresponding: pshiao@msn.com

Supplementary Table S1. Comparisons on demographic/environmental factors per race groups.

Parameters		Asian (N = 40) n (%)	Caucasian (N = 34) n (%)	Hispanic (N = 23) n (%)	African American (N = 9) n (%)	*p*
Gender	Male	13 (32.5%)	15 (44%)	8 (35%)	3 (33%)	0.76
	Female	27 (67.5%)	19 (56%)	15 (65%)	6 (67%)	
Age, Years	M ± SD	53 ± 14	58 ± 16	50 ± 17	54 ± 20	0.33
	(range)	(19 – 75)	(21 – 80)	(18 – 75)	(19 – 77)	
BMI status	Obese	0 (0%)	10 (29%)	12 (52%)	4 (44%)	<0.0001
Alcohol drinker	Yes	15 (38%)	27 (79%)	13 (57%)	2 (22%)	0.0007
Smoker	Yes	4 (10%)	1 (3%)	3 (13%)	1 (11%)	0.54
Total Polymorphism	≥4	21 (53%)	16 (47%)	5 (22%)	1 (11%)	0.02

Note: BMI: body mass index.

Supplementary Table S2. Comparisons on dietary parameters: Healthy Eating Index per race groups.

Parameters, unit	**Asian** (N = 40) M ± SD	**Caucasian** (N = 34) M ± SD	**Hispanic** (N = 23) M ± SD	**African American** (N = 9) M ± SD	*p*
Calorie, per day	1811 ± 1062	1566 ± 761	1520 ± 910	1241 ± 473	0.29
Total Fruit, 0.8 cups	2.3 ± 1.9	1.3 ± 0.9	1.0 ± 0.9	1.1 ± 0.6	0.0009
Whole Fruit, 0.4 cups	1.6 ± 1.3	1.0 ± 0.9	0.83 ± 0.8	0.8 ± 0.6	0.006
Vegetables, 1.1 cups	1.7 ± 1.1	1.7 ± 1.1	1.5 ± 1.5	0.7 ± 0.6	0.13
Dark green, 0.4 cups	0.9 ± 0.6	1.0 ± 0.7	0.9 ± 0.8	0.4 ± 0.5	0.21
Total Grain, 3 oz	5.7 ± 3.5	3.8 ± 2.4	4.5 ± 2.8	3.9 ± 2.4	0.07
Whole Grain, 1.5 oz	1.9 ± 2.1	1.8 ± 1.3	1.3 ± 1.3	1.8 ± 1.6	0.62
Dairy, 1.3 cups	1.4 ± 0.5	1.0 ± 0.5	2.3 ± 0.7	1.1 ± 1.1	0.48
Protein, 2.5 oz	6.5 ± 5.2	5.5 ± 2.8	5.9 ± 4.2	3.2 ± 2.3	0.19
Oil and nuts, 12 g	38 ± 24	36 ± 18	38 ± 24	27 ± 13	0.59
Saturated Fat, g, 8%	18 ± 11	20 ± 14	18 ± 10	15 ± 6.0	0.53
Sodium, ≤1.1 g	3.8 ± 2.3	2.8 ± 1.3	3.0 ± 2.0	2.0 ± 0.9	**0.03**
Empty Calorie, 19%	325 ± 214	393 ± 272	331 ± 196	349 ± 134	0.61
HEI score	78 ± 8.2	75 ± 9.4	74 ± 9.6	71 ± 9.8	0.07
HEI score ≥77	24 (60%)	18 (54%)	10 (44%)	2 (22%)	0.18
HEI score ≥80	20 (50%)	11 (32%)	6 (26%)	2 (22%)	0.15

Note: M: mean, SD: standard deviation, oz: ounce, g: gram, HEI: Healthy Eating Index

Supplementary Table S3. Comparisons on dietary parameters: Recommended daily intakes per race groups.

Parameters, RDI		**Asian** (N = 40) n (%)	**Caucasian** (N = 34) n (%)	**Hispanic** (N = 23) n (%)	**African Americar** (N = 9) n (%)	*p*
Carbohydrates (45-65%)	≥45%	32 (85%)	19 (56%)	15 (65%)	7 (78%)	0.04
Protein (10-35%)	≥20%	17 (423%)	14 (41%)	6 (26%)	1 (11%)	0.21
Total Fat (20-35%)	≥35%	6 (15%)	16 (47%)	12 (52%)	3 (33%)	0.007
Saturated Fat (<10%)	≥10%	14 (35%)	21 (62%)	13 (57%)	7 (78%)	0.037
Cholesterol, <300 mg	<100%	25 (63%)	27 (79%)	18 (78%)	8 (89%)	0.22
Sodium, <2300 mg	<100%	15 (38%)	13 (38%)	7 (30%)	5 (56%)	0.64
Fiber, ≥25 g	≥100%	8 (20%)	4 (12%)	3 (13%)	1 (11%)	0.75
Total Folate, 400 mcg	≥100%	17 (43%)	9 (27%)	4 (17%)	4 (44%)	0.15
Vitamin B1, 1.1 mg	≥100%	27 (68%)	21 (62%)	13 (57%)	4 (44%)	0.58
Vitamin B2, 1.1 mg	≥100%	32 (80%)	26 (77%)	15 (65%)	5 (56%)	0.34
Vitamin B6, 1.3 mg	≥100%	30 (75%)	23 (68%)	12 (52%)	3 (33%)	0.061
Vitamin B12, 2.4 mcg	<150%	14 (35%)	14 (41%)	11 (48%)	5 (56%)	0.61
Niacin, 14 mg	≥100%	19 (48%)	15 (44%)	5 (22%)	4 (44%)	0.22
Calcium, 1,000 mg	≥100%	22 (55%)	17 (50%)	8 (35%)	3 (33%)	0.36
Magnesium, 320 mg	≥75%	19 (48%)	21 (62%)	8 (35%)	4 (44%)	0.24
Iron, 8 mg	≥100%	20 (50%)	10 (29%)	10 (44%)	4 (44%)	0.35
Zinc, 8 mg	≥100%	23 (58%)	17 (50%)	11 (48%)	2 (22%)	0.29
Methionine, 13 mg/Kg	<150%	11 (28%)	15 (44%)	13 (57%)	6 (67%)	0.052

Note: RDI: recommended daily intake, g: gram; mg: milligram, mcg: microgram, Kg: Kilogram.

Supplementary Table S4. Major dietary parameters as predictors of colorectal cancer.

**Term**	**Number of Splits**	**G²**	**Column Contributions**	**Portion**
Total Vegetable 10oz	46	1.29		0.17
Total Folate 100%	48	1.24		0.16
Vitamin B12 150%	45	1.22		0.16
Total Grains 4oz	46	1.01		0.13
HEI 77	57	0.97		0.13
Milk Soy 6oz	51	0.72		0.09
Fiber 16g	44	0.60		0.08
Thiamin 100%	38	0.51		0.07

Note: HEI: Healthy Eating Index (median score 77).

Supplementary Table S5. Gene-diet interactions on the predictors of colorectal cancer: Baseline logistic regression model and generalized regression Elastic Net models.

	Logistic Regression with Validation		Generalized Regression Elastic Net Model			
			AICc Validation		Leave-One-Out Validation	
Parameters	Estimate (95% CI)	p (X^2)	Estimate (95% CI)	p (X^2)	Estimate (95% CI)	p (X^2)
(Intercept)	-0.77 (-2.57, 1.03)	0.40	-0.84 (-2.4, 0.68)	0.28	-0.78 (-2.2, 0.6)	0.27
Thiamin * HEI	-3.6 (-6.5, -0.8)	0.01	-2.9 (-5.0, -0.68)	0.01	-2.6 (-4.7, -0.48)	0.02
Gender * BMI Overweight	-2.2 (-4.7, 0.25)	0.08	-2.8 (-4.8, -0.73)	0.008	-2.1 (-3.5, -0.67)	0.004
Gender	2.0 (0.21, 3.8)	0.03	2.4 (1.1, 3.7)	0.0004	2.1 (1.0, 3.3)	0.0001
Total Polymorphism	-1.0 (-2.3, 0.26)	0.12	-1.5 (-2.6, -0.36)	0.01	-1.5 (-2.6, -0.48)	0.004
HEI	2.7 (0.46, 5.0)	0.02	2.60 (0.52, 4.7)	0.02	2.6 (0.63, 4.6)	0.01
Thiamin	1.8 (0.002, 3.6)	0.0497	1.8 (0.36, 3.2)	0.01	1.6 (0.27, 2.9)	0.02
Age	-1.3 (-2.5, -0.09)	0.04	-1.4 (-2.4, -0.43)	0.005	-1.2 (-2.1, -0.29)	0.01
Vegetable 10 oz	0.91 (-0.35, 2.2)	0.16	0.85 (-0.22, 1.9)	0.12	0.86 (-0.13, 1.8)	0.09
BMI Overweight	0.30 (-1.2, 1.8)	0.69	0.48 (-0.87, 1.8)	0.49	0 (0, 0)	1.00
Misclassification Rate	0.24		0.24		0.22	
AICc	63		129		n/a	
Area under the curve	0.82		0.84		0.85	

Note. CI: confidence interval; HEI: Health Eating Index score, AICc: Akaike's information criterion with correction.

Supplementary Table S6. Baseline logistic regression and generalized regression Elastic Net on the selected predictors of colorectal cancer from genetic and dietary factors.

Parameters	Logistic Regression Original model with Validation				Generalized Regression Elastic Net						
					AICc Validation				Leave-One-Out Validation		
	p (X^2)	MR	AICc	AUC	*p* (X^2)	MR	AICc	AUC	*p* (X^2)	MR	AUC
5 Factors, 2 interactions											
Thiamin * HEI	0.04	0.30	61	0.82	0.02	0.26	136	0.79	0.02	0.26	0.81
Gender * BMI	0.16				0.02				0.04		
Gender	0.02				0.0001				<0.0001		
Total gene PM	0.09				0.007				0.0045		
HEI	0.047				0.011				0.01		
Thiamin	0.09				0.04				0.04		
BMI	0.65				0.46				0.67		
6 Factors, 2 interactions											
Thiamin * HEI	0.02	0.2	59	0.85	0.013	0.25	129	0.83	0.009	0.27	0.84
Gender * BMI	0.10				0.012				0.04		
Gender	0.02				0.0003				<0.0001		
Total gene PM	0.15				0.012				0.007		
HEI	0.03				0.014				0.007		
Thiamin	0.06				0.02				0.007		
BMI	0.61				0.39				0.36		
**Age**	0.03				0.004				0.002		
7 Factors, 2 interactions (Supplementary Table 5)											
Thiamin * HEI	0.01	0.24	63	0.82	0.01	0.24	129	0.84	0.02	0.22	0.85
Gender * BMI	0.079				0.008				0.004		
Gender	0.028				0.0004				0.0001		
Total gene PM	0.12				0.0095				0.004		
HEI	0.012				0.02				0.0097		
Thiamin	0.0497				0.01				0.02		
BMI	0.69				0.49				1.00		
Age	0.035				0.0048				0.0097		
**Vegetable**	0.16				0.12				0.088		
8 Factors, 3 interactions (Table 5)											
Thiamin * HEI	0.013	0.22	71	0.85	0.012	0.25	130	0.85	0.014	0.21	0.86
Gender * BMI	0.07				0.001				0.0003		
Gender	0.04				0.0005				<0.0001		
Total gene PM	0.15				0.01				0.004		
HEI	0.02				0.02				0.015		
Thiamin	0.06				0.03				0.011		
BMI	0.71				0.68				1.00		
Age	0.04				0.003				0.005		
**Vegetable**	0.09				0.07				0.04		
***MTHFR*677*BMI**	0.29				0.06				0.10		
***MTHFR*677**	0.48				0.33				0.81		
9 Factors, 3 interactions											
Thiamin * HEI	0.008	0.24	76	0.85	0.0098	0.23	129	0.86	0.019	0.22	0.87
Gender * BMI	0.04				0.001				0.0003		
Gender	0.053				0.0003				<0.0001		
Total gene PM	0.26				0.03				0.02		
HEI	0.01				0.02				0.02		
Thiamin 100%	0.24				0.15				0.14		
BMI	0.82				0.80				1.00		
Age	0.023				0.0014				0.0006		
Vegetable	0.04				0.03				0.09		
*MTHFR*677*BMI	0.30				0.06				0.07		
MTHFR 677	0.27				0.26				0.40		
**Folate**	0.09				0.10				0.18		

Note: MR – Misclassification rate; AICc – Akaike's information criterion with corrections; AUC – Area under the curve; HEI: Healthy Eating Index, ≤ or >77; BMI: body mass index, overweight or not; Total gene PM: polymorphism total score < or ≥ 4 (0-10 range), Thiamin 100% or not of recommended daily intake (RDI); Age: ≤ or > 56; Vegetable: 10 oz intake or not; Folate: 100% RDI or not.

Prediction Profiler

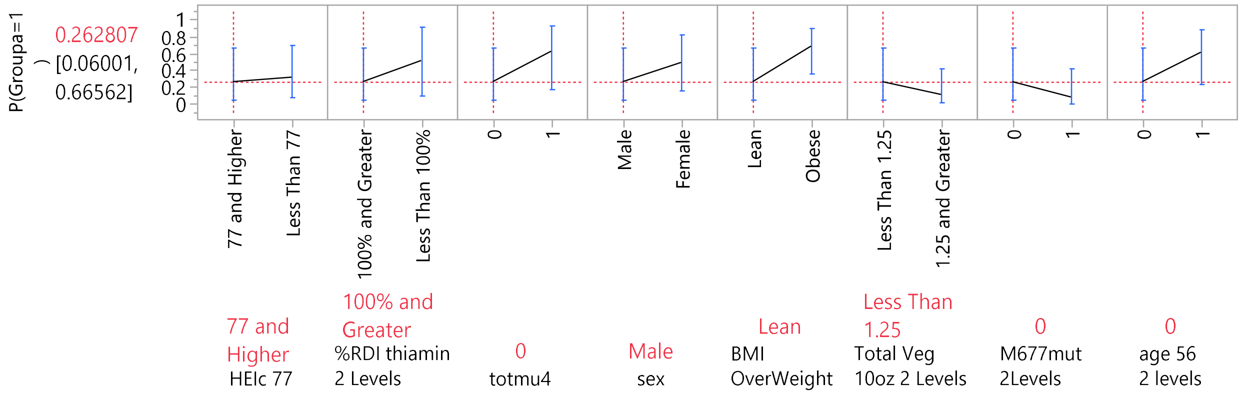

Interaction Profilers

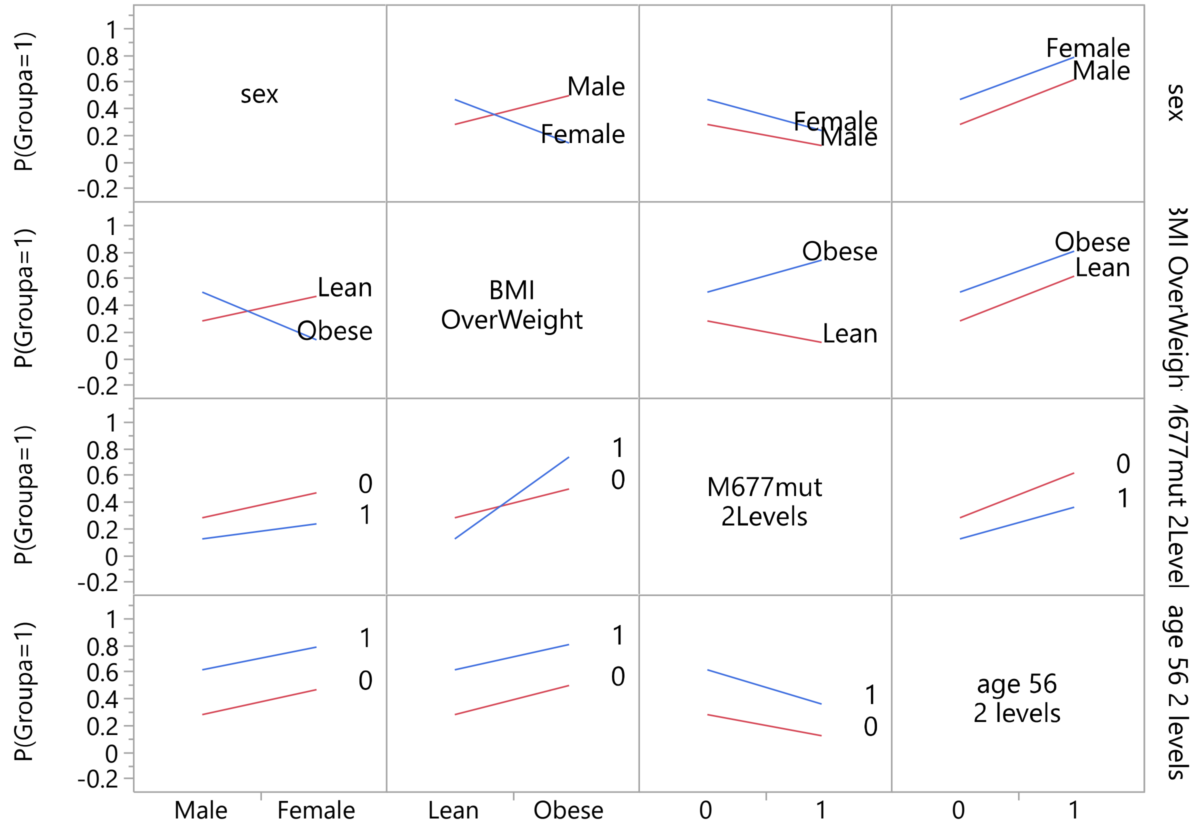

Supplementary Figure S1. Gene-diet Interaction: (a). prediction profiler, interaction profiles - gender interacting with overweight per body mass index (BMI), *MTHFR* 677 polymorphism interacting with overweight based on BMI. Note. p(Groupa) = 1 is the probability of predicting a level 1 (cancer status versus 0 as control status) response.

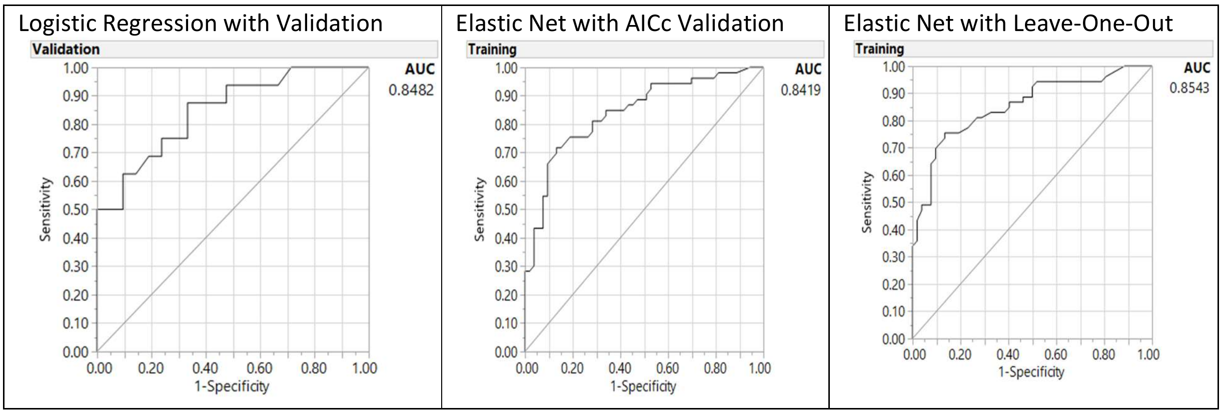

Supplementary Figure S2. Area under the receiver operating characteristic curve (AUC) for baseline logistic regression model (left panel), Elastic Net with Akaike's information criteria with correction (AICc) validation model (middle) and Leave-One-Out validation model (right panel) on the predictors of colorectal cancer.

Supplementary File -

Consent Form (Template)

California State Committee for the Protection of Human Subjects, CPHS-12-12-1007, 2013-2019

Informed Consent Form (Cancer Patient)

Official Title of Project: Colorectal Cancer Family Epigenetic Study Program
Principal Investigator: S. Pamela Shiao, PhD, MSN, RN, FAAN
Institution Conducting the Research: Azusa Pacific University

1. Purpose, Participation, and Procedures of the study

    Purpose: The study for which you are being asked to participate is designed to look into different variations of human genes and risk factors that have been found to be associated with colorectal cancer for various race-ethnicity groups, for cancer prevention.

    Participation and Procedure: To be a voluntary participant in this study, you and a family member who lived with you while you were a colorectal cancer patient will be asked detailed questions in an interview at your home about environment and health, family health history, and dietary intake; and have your blood drawn for analyses of nutrient levels and common genes that are known to be related to the development of colorectal cancer. The study asks that you grant the researchers permission to view your medical or clinical record for your health history through the databases already submitted to the California Cancer Registry. This first home visit will last about 1.5 hours. A second visit, which will last about 30 minutes, will occur about 3 months later to follow up the changes of environment and health status.

2. Description of Risks to the Participants

    You may feel some minor pain or fainting with blood draw(s), or bruises after blood draw(s), which will be done by RNs who have previous training and clinical experiences. Every effort will be made to minimize any discomfort or pain. If you feel faint, you may lie down. If you feel any discomfort, please tell the researcher whether you want to continue, take a break, or have some water. Finding out the results of your blood tests could raise some concerns or cause distress; if so, please discuss those concerns with your personal physician. You could feel some discomfort in answering interview questions that might seem embarrassing. There is also the possibility of accidental disclosure of your private information despite efforts of the researchers to prevent this.

3. Description of the Measures to be Taken to Ensure Participant Confidentiality

    Any information that is obtained in connection with this study and that can be identified with you will remain confidential. The California Cancer Registry (CCR) prohibits sharing personal identity data with any other parties for any legal reasons or purposes, so your identity cannot be disclosed from this study by the research team. A code number will be assigned to your data, and your name will be removed from the data. Your identity information will be stored separately from your de-identified data in a locked cabinet. When the results of the research are published or discussed in conferences, no information will be included that would reveal your identity. Only the research team and funding agencies can access your research data in accordance with regulations for research. Although every effort will be made to keep the research data confidential, DNA data is itself highly personal. While the researchers will not reveal your name in connection with your research data, there is research showing that personal identity may be found from DNA data.

4. Description of any Benefits to Participants from participation in this Study

You are entitled to know the results of your blood tests and nutrient levels, which can be sent to your personal physician, and might benefit from knowledge of your nutrient levels and personal genome information. Other than the blood results and nutrient levels, you may not receive any direct benefits from participating in this study; however, your participation will help improve the knowledge about genetic factors and environmental factors for colorectal cancer prevention. Your participation may also benefit other people with similar concerns for cancer prevention in the future.

5. Alternative Procedures or Treatments to this Study

This is research and does not have anything to do with the current services you are receiving. You can withdraw from the study at any time without penalty by calling Dr. Pamela Shiao, the Principal Investigator, at 818-233-6112.

6. Compensation for Participant related to this Study

Other than the knowledge gained from this study, there is no compensation from participation.

7. Treatment for Injury related to this Study

If you have a medical emergency during the study you may contact your own doctor, or seek treatment at the care centers available to you. Be sure to tell the doctor or his/her staff that you are in a research study being conducted at your home setting in collaboration with Azusa Pacific University (APU). If needed, ask them to call Dr. Pamela Shiao, the Principal Investigator, at 818-233-6112, for information or clarification related to your care.

In the event of any physical injury resulting from research procedures, you will not be provided medical treatment through APU, however, you may seek treatment with your primary care physician or referral centers for genetic testing. APU will not provide you with financial compensation if you are injured in this study.

8. Potential Conflict of Interest of the Researchers

Neither the Principal Investigator nor anyone else involved in this research has any financial or other conflict of interest.

9. Contacts for Participant Questions about this study

If you have questions regarding your participation in this research study or have any questions about your rights as a research subject, please contact the Principal Investigator using the phone number listed above in item 7.

10. Voluntary Participation in this Study

Your participation is voluntary which means you can choose whether or not you want to participate. You may withdraw any time without penalty. If you decline to continue, any data gathered to that point may be used in data analysis. If you choose not to participate, there will be no loss of benefits to which you are entitled.

11. Research Participant's Bill of Rights Provided to Participants

This document explains your rights as a research subject. You have been asked to participate as a subject in a study. Before you decide whether you want to participate in the study, you have a right to: a) be informed of the nature and purpose of the study, b) be given an explanation of what will happen during the study and of how you are expected to participate, c) be given an explanation of

any risks or discomforts that may be experienced as a result of participating in the study, d) be given an explanation of any benefits that you may receive from participation in the study, e) be told of other appropriate choices that may be better or worse than being in the study, and be told of the risks and benefits of those other choices, f) have the opportunity to ask questions about the study or about your participation in it, both before agreeing to participate in the study and during the course of the study, g) be told that you may withdraw your consent and participation in the study at any time, and that your withdrawal will not affect your services, h) be told that you may refuse to answer any question, i) be given a copy of the signed and dated consent form, j) be free of pressure when considering whether to consent to, and participate in, the study, and k) be informed, upon request, about the results of the study. Concerning your rights or treatment as a research subject, you may contact the Research Integrity Officer at Azusa Pacific University (APU) at (626) 812-3034.

12. Consent Statement and Signature

I give my consent to participate in this research and have received a copy of this consent form, a California Cancer Registry brochure, and the Research Participant's Bill of Rights.
.

____________________	____________________	____________________
Printed Name of Participant	Signature of Participant	Date Signed
____________________	____________________	____________________
Printed Name of Representative	Signature of Representative	Date Signed
